# Effect of PEEP on inspiratory resistance components in patients with acute respiratory distress syndrome ventilated at low tidal volume

**DOI:** 10.5935/0103-507X.20190071

**Published:** 2019

**Authors:** Sebastian Fredes, Emilio Steinberg, Norberto Tiribelli, Analia Santa Maria, Mariana Berté, Nicolás Segura, Diego Noval, Santiago Ilutovich

**Affiliations:** 1 Unidad de Cuidados Intensivos, Sanatorio La Trinidad Mitre - Buenos Aires, Argentina.

**Keywords:** Respiratory distress syndrome, adult, Continuous positive airway pressure, Respiration, artificial

## Abstract

**Objective:**

To describe the behavior of inspiratory resistance components when positive end-expiratory pressure (PEEP) increases in patients with acute respiratory distress syndrome under a protective ventilation strategy.

**Methods:**

In volume-controlled mode, at 6mL/kg and constant flow, end-inspiratory occlusions were performed at 0, 5 10, 15 and 20cmH_2_O PEEP. Peak, initial and plateau pressure values were assessed, calculating the maximum, minimum and differential resistances. The results were compared by repeated measures analysis of variance (ANOVA) with post hoc Bonferroni correction, considering p < 0.05 significant.

**Results:**

The highest maximum resistance was observed at the lowest PEEP levels. The values for 10 and 15cmH_2_O PEEP significantly differed from those for 5 and 0cmH_2_O PEEP, whereas that for 20cmH_2_O PEEP only significantly differed from that for 0cmH_2_O PEEP (p < 0.05). The minimum resistance behaved similarly to the maximum resistance; the values for PEEP levels from 10cmH_2_O to 20cmH_2_O significantly differed from those for 0 and 5cmH_2_O PEEP (p < 0.05). Differential resistance showed the opposite variation to the maximum and minimum resistances. The only PEEP level that showed significant differences from 0 and 5cmH_2_O PEEP was 20cmH_2_O PEEP. Significant differences were also found between 15 and 5cmH_2_O PEEP (p < 0.05).

**Conclusions:**

During protective ventilation in patients with acute respiratory distress syndrome, the maximum resistance of the respiratory system decreases with PEEP, reflecting the minimum resistance response, whereas differential resistance increases with PEEP.

## INTRODUCTION

Basic monitoring of patients under mechanical ventilation (MV) in volume-controlled continuous mandatory ventilation (VC-CMV) mode with constant flow is an invaluable diagnosis, treatment and follow-up tool. Knowledge and experience in interpreting respirator graphics allow a personalized approach to the patient. This monitoring makes it possible to classify the state of the respiratory system according to its modifications in elastance and resistance (Rrs).

**Figure 1:**
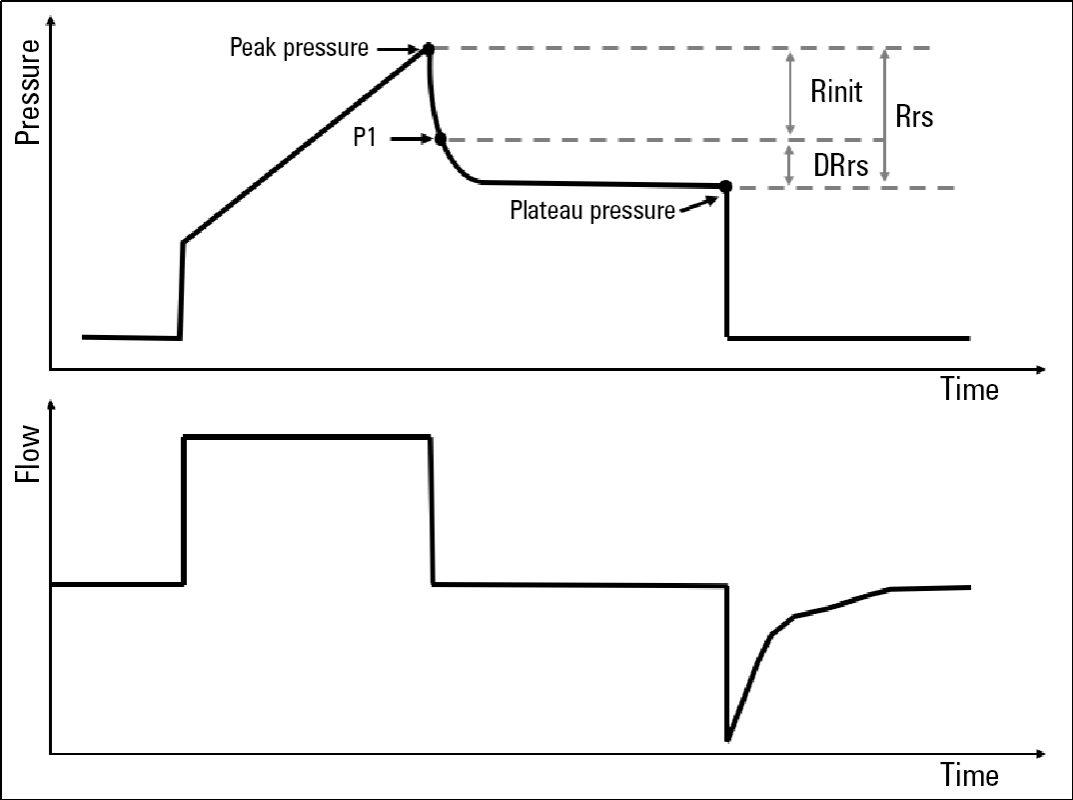
Graph of pressure and flow as a function of time showing the maximum airway pressure during the inspiratory cycle, the pressure at first flow F = 0 or initial pressure, and the pressure after a 2-second end-inspiratory pause (plateau pressure). The maximum resistance is calculated as the difference between peak pressure and plateau pressure divided by inspiratory flow. The minimum resistance is calculated as the difference between peak pressure and initial pressure divided by maximum inspiratory flow, and differential resistance is calculated as the difference between the maximum and minimum resistances. Rinit - minimum resistance; DRrs - differential resistance; Rrs - maximum resistance.

Acute respiratory distress syndrome (ARDS) is an acute-onset respiratory condition that presents with hypoxemia, bilateral opacities on chest X-ray and changes in the mechanics of the respiratory system.^(1)^ The elastance of the respiratory system has been the focus of considerable research, whereas Rrs has been mostly overlooked. Several methods for assessing Rrs have been described, including the "rapid airway occlusion technique", which consists of performing a 2-second end-inspiratory pause and observing pressure changes under constant flow. This abrupt flow interruption will generate a drop in system pressure. This pressure has biphasic morphology: a sharp decrease in pressure, whose variation occurs between the peak (Ppeak) and initial (P1) pressures in the first phase, is followed by a00 more gradual decrease in pressure in the second phase. In this case, the change in pressure occurs between P1 and the pressure after a 2-second end-inspiratory pause (Pplat). The first is termed "minimum resistance" (Rinit): because the inertia of the gas is negligible, the initial decrease in pressure after interrupting the flow is exclusively attributed to its friction with the airway. The second is termed "differential resistance" (DRrs), which is linked to stress relaxation and the pendelluft phenomenon typical of lung heterogeneity. The first is a condition of the materials, related to the temporal dependence of mechanical measures: over time, the energy required to maintain a deformed system decreases, which applies to the respiratory system. In turn, pendelluft explains the gas redistribution inside the lung in the absence of flow, which increases under heterogeneous conditions. However, these 2 phenomena are apparently indistinguishable from each other (Figure 1). The ratio of ∆ pressure to flow is Rrs (Rrs = ∆P/F).^(2)^ In ARDS, the increase in Rrs can be attributed to alveolar flooding, loss of lung volume, vagal reflex and bronchial hyperreactivity.^(3)^

Several studies have assessed positive end-expiratory pressure (PEEP) effects on Rrs in ARDS. They showed that Rrs increased with PEEP, as a result of the increase in DRrs due to a possible overdistension of ventilated units, to an increase in lung heterogeneity, or to both.^(4-6)^

The objective of the present study was to describe the behavior of different inspiratory resistance components when PEEP increases in patients with moderate/severe ARDS under ventilation with a low tidal volume (TV) strategy.

## METHODS

The measurements were conducted at the intensive care unit (ICU) of the *Sanatorio de La Trinidad Mitre*, Autonomous City of Buenos Aires, from October 2015 to September 2017.

A cross-sectional, descriptive, retrospective study was conducted.

Patients under MV older than 18 years old who met ARDS diagnostic criteria according to the Berlin definition^(1)^ at the beginning of MV or as a complication were included in the study. Pregnant patients and those with limited therapeutic effort, history of neuromuscular disease or bronchopleural fistula or unable to use an esophageal balloon were excluded from the study.^(7)^

Demographic data of the participants were collected, in addition to severity scores, oxygenation rates, ventilatory monitoring variables, reasons for MV, and outcome variables.

Evita XL (Dräger, Lübeck, Germany) and Maquet Servo I and S (Solna, Sweden) ventilators, with low ventilator circuit compliance (1.5mL/cmH_2_O), were used. Before connecting the patient to MV, the ventilator was checked for compressible volume compensation and circuit resistance, in addition to testing the proportional valves, inspiratory and expiratory modules, flow and pressure sensors. Data were acquired using a respiratory mechanics monitor (FluxMed, MBMed, Bs.As., Argentina) to measure airway flow and pressure (Paw) with a fixed-orifice differential pressure sensor connected to the endotracheal tube or tracheostomy tube of the patient. The volume was calculated by integrating flow over time. Flow and Paw signals were acquired simultaneously using specialized software (FluxReview, MBMed, Bs.As., Argentina). Before starting the measurements, the patient was aligned and semiseated at 45º. Subsequently, the endotracheal tube cuff was controlled, followed by aspiration of secretions if necessary. After the inclusion, the ventilator was set to VC-CMV mode, adjusting the TV to 4 to 6mL/kg predicted body weight, with a constant flow of 60L/min, 10cmH_2_O PEEP and previous FiO_2_. In all patients included in the study, esophageal pressure (Pes) was used as a guide to set the MV. Its correct positioning was confirmed using the dynamic occlusion method,^(8)^ confirming the absence of respiratory effort by inspecting the curve. Subsequently, the PEEP was set to 20cmH_2_O, gradually decreasing from 20 to 0cmH_2_O, in 4 5cmH_2_O steps. The duration of each step was 10 minutes (totaling 50 minutes). At the end of each step, end-inspiratory pause maneuvers were performed using the function provided in the ventilators used. An offline observational analysis of Paw, flow and esophageal pressure signals from the respiratory mechanics monitor was performed. At each PEEP level, Ppeak, P1, and plateau (Pplat) pressures, total PEEP (PEEPtot) and end-inspiratory (Pesi) and end-expiratory (Pese) esophageal pressure values were recorded, calculating the end-inspiratory (Ptpi) and end-expiratory (Ptpe)^(7)^ transpulmonary pressures, which were defined as follows:

- Ppeak = highest Paw value during the inspiratory cycle;
- P1 = Paw at first flow F = 0 at the beginning of the inspiratory pause;
- Pplat = Paw after a 2-second end-inspiratory pause;
- PEEPtot = Paw after a 2-second end-expiratory pause;
- Pesi = Pes after a 2-second end-inspiratory pause;
- Pese = Pes after a 2-second end-expiratory pause;
- Ptpi = Difference between Pplat and Pesi; and
- Ptpe = Difference between PEEPtot and Pese.

Rrs was calculated as the difference between Ppeak and Pplat divided by inspiratory flow (Rrs = PPeak - Pplat/F), Rinit was calculated as the difference between Ppeak and P1 divided by inspiratory flow (Rinit = PPeak - P1/F), and DRrs was calculated as the difference between P1 and PPlat divided by flow (DRrs = P1 - Pplat/F) (Figure 1).

To observe the behavior of the elastic component of the respiratory system when changing the PEEP, the elastance of the respiratory system was calculated as the difference between Pplat and total PEEP divided by TV, lung elastance was calculated as the difference between inspiratory and expiratory transpulmonary pressures divided by TV, and chest wall elastance was calculated as the difference between Pesi and Pese divided by TV.

The study was approved by the Research and Education Committee of the Hospital, under record number F004-01-A(01)2018. Considering its retrospective nature, informed consent was not required. Data confidentiality was preserved by creating a coded registration form for each participant. Names or any data that could identify the participants will be kept under extreme confidentiality, and in no case will the participants' identities be made public.

### Statistical analysis

Descriptive analysis of the variables was performed. The values expressed as the mean and standard deviation or median and interquartile range depending on the distribution of the numerical variables or as numbers and percentages for qualitative variables. The results were compared by repeated measures analysis of variance (ANOVA) with post hoc Bonferroni correction. Different PEEP levels were analyzed as previously reported in the literature: medium-to-high (10, 15 and 20cmH_2_O) and low (0 and 5cmH_2_O). A value of p < 0.05 was considered significant.

## RESULTS

In total, 24 patients, including 13 men, with moderate-to-severe ARDS (131.2 ± 32.4 PaFiO_2_ on inclusion) according to the Berlin Definition^(1)^ were included in the study (Table 1). The patients were ventilated in VC-CMV mode with 382.8mL mean TV, representing 5.8mL/kg predicted body weight, 27 breaths per minute mean respiratory rate (RR) and 0.99L/sec V. In addition, 54.1% patients were extubated, with 33.3% ICU mortality.

**Table 1:** Characteristics of the patients included in the study

Characteristics of the patients	
N	24
Male sex	13/24 (54.16)
SAPS II	46.9 ± 14.78
PaO_2_/FiO_2_ on inclusion	131.2 ± 32.45
Moderate ARDS	22 (91.66)
Severe ARDS	2 (8.34)
Reason for MV	
CRD	1 (4.16)
ARF	19 (79.16)
Pneumonia	4 (16.60)
Postoperative	4 (16.60)
Sepsis	3 (12.50)
Trauma	3 (12.50)
ARDS	1 (4.16)
Aspiration	1 (4.16)
APE	1 (4.16)
CRA	1 (4.16)
Other	1 (4.16)
Coma	3 (12.50)
Neuromuscular disease	1 (4.16)
Ventilator settings	
TV (mL)	382.8 ± 85.47
TV (mL/kg)	5.81 ± 0.80
RR	27.04 ±4.98
Flow	0.99 ± 0.06
Inspiratory time	0.75 ± 0.12
Days of MV	8 [4.5 - 12.5]
Extubated	13/24 (54.10)
Reintubated	3/13 (23)
Tracheostomized	6/24 (25)
Days of ICU	11 [7 - 19.70]
ICU mortality	8/24 (33.33)

SAPS II - Simplified Acute Physiology Score II; PaO_2_/FiO_2_ - ratio of the partial pressure of arterial oxygen to the fraction of inspired oxygen; ARDS - acute respiratory distress syndrome; MV - mechanical ventilation; CRD - chronic respiratory disease; ARF - acute respiratory failure; APE - acute pulmonary edema, CRA - cardiorespiratory arrest; TV - tidal volume; RR - respiratory rate; ICU - intensive care unit.

**Figure 2:**
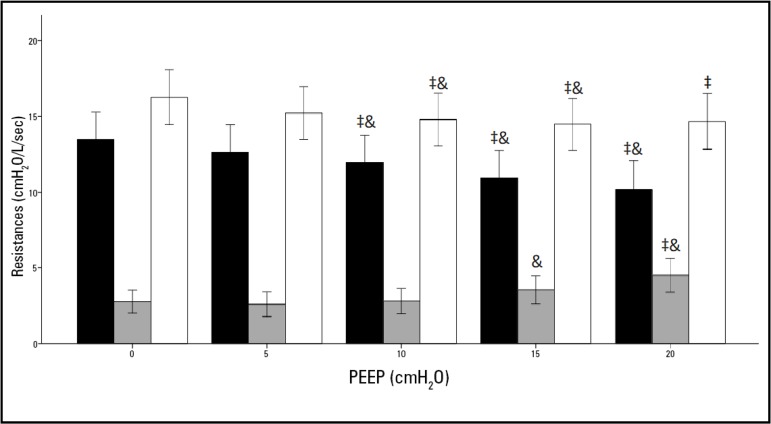
Variations in the maximum (white bars), minimum (gray bars) and differential (black bars) resistances of the respiratory system with different of positive end-expiratory pressure levels; post hoc test with Bonferroni correction (‡ *versus* 0cmH_2_O PEEP, < 0.05, & *versus* 5cmH_2_O PEEP, < 0.05). PEEP - positive end-expiratory pressure.

The behaviors of Rrs, Rinit and DRrs are outlined in table 2 and in figure 2.

**Table 2:** Average variation in resistive pressure differences, resistances and static mechanical variables of the respiratory system at different PEEP levels; analysis of variance (ANOVA)

	PEEP 0	PEEP 5	PEEP 10	PEEP 15	PEEP 20
Ppeak - Pplateau (cmH_2_O)	16.03 (4.06)	15.07 (3.82)	14.64 (3.77)‡&	14.3 (3.83)‡&	14.38 (3.83)‡
Ppeak- P1 (cmH_2_O)	13.41 (4.13)	12.56 (4.18)	11.94 (4.08)‡&	10.99 (4.24)‡&	10.02 (4.41)‡&
P1 - Pplateau (cmH_2_O)	2.64 (1.69)	2.5 (1.9)	2.69 (1.96)	3.3 (2.06)&	4.36 (2.39)‡&
Rrs (cmH_2_O/L/sec)	16.33 (4.47)	15.33 (4.26)	14.91 (4.24)‡&	14.54 (4.2)‡&	14.65 (4.36)‡
Rinit (cmH_2_O/L/sec)	13.61 (4.36)	12.76 (4.41)	12.13 (4.29)‡&	11.15 (4.39)‡&	10.14 (4.52)‡&
DRrs (cmH_2_O/L/sec)	2.72 (1.84)	2.56 (1.99)	2.77 (2.07)	3.38 (2.15)&	4.5(2.66)‡&
Ers (cmH_2_O/L)	36.53 (16.91)	31.43 (15.01)	31.31 (15.84)	34.54 (20.59)	38.16 (16.8)
Ecw (cmH_2_O/L)	9.54 (5.21)	8.34 (5.59)	7.78 (4.49)	7.41 (4.62)	7.81 (4.46)
El (cmH_2_O/L)	26.98 (16.87)	23.08 (16.43)	23.52 (17.24)	27.12 (21.74)	30.34 (17.67)

PEEP - positive end-expiratory pressure; Ppeak - peak pressure; Pplateau - plateau pressure; P1 - initial pressure; Rrs - maximum resistance; Rinit - minimum resistance; DRrs - differential resistance; Ers - elastance of the respiratory system; Ecw - chest wall elastance; El - lung elastance.

& p < 0.05 *versus* 5cmH_2_O PEEP.

‡ p < 0.05 *versus* 0cmH_2_O PEEP.

The highest Rrs was observed at the lowest PEEP levels. Values at 10 and 15cmH_2_O PEEP significantly differed from those at 5 and 0cmH_2_O PEEP, whereas values at 20cmH_2_O PEEP only significantly differed from values at 0cmH_2_O PEEP (p < 0.05).

Rinit showed the same behavior as has Rrs. Higher values were observed at lower PEEP levels. For 10cmH_2_O PEEP and 20cmH_2_O PEEP, all variables showed significant differences from 0 and 5cmH_2_O PEEP (p < 0.05).

Differential resistances showed the opposite behavior to Rrs and Rinit, with lower DRrs at lower PEEP levels. In this case, only values at 20cmH_2_O PEEP were significantly different from those at 0 and 5cmH_2_O PEEP. Significant differences also occurred between 15cmH_2_O PEEP and 5cmH_2_O PEEP (p < 0.05).

Elastance of the respiratory system increased at high PEEP levels (15 and 20cmH_2_O), similarly to lung elastance, albeit with nonsignificant differences (Table 2).

## DISCUSSION

The objective of this study was to assess the effect of PEEP on different inspiratory resistance components in patients with RDS under protective ventilation. We found that when using a low TV strategy, increases in PEEP decreased Rinit and increased DRrs. Because these responses had different magnitudes, Rrs decreased.

Maximum resistance depends on 2 components: nonelastic or flow resistance and elastic resistance corresponding to the pendelluft phenomenon, associated with heterogeneous expiratory time constants of the lung and to the stress relaxation phenomenon.

The lower TV ventilation observed in the acute lung injury (ALI)/acute respiratory distress syndrome (ARDS) (ARMA) trial,^(9)^ published over 20 years ago, changed the ventilation strategy for patients by demonstrating that a TV of 6mL/kg predicted weight, compared with 12mL/kg, decreased mortality. Evidence of changes in Rrs with changes in PEEP in patients with ARDS precedes such studies; therefore, we assume that the ventilatory strategy used at that time was high TV.

In 1991, Pesenti et al.^(4^ assessed the effect of PEEP on the respiratory resistance component by comparing 21 healthy subjects with 11 patients with ARDS according to criteria from a previous study by Gattinoni et al.^(10^ The 21 subjects without lung disease were subjected to 3 PEEP levels (0, 5 and 10cmH_2_O), whereas patients with ARDS were subjected to 5 PEEP levels (0, 5, 10, 15, 20cmH_2_O). In addition to differences from healthy subjects, the study showed that Rrs increased as the PEEP level increased, reflecting the behavior of DRrs, with significant differences when comparing 10cmH_2_O PEEP with 15 and 20cmH_2_O PEEP. The authors explained that high PEEP levels, in highly heterogeneous lungs, could increase stress relaxation and that PEEP could cause overdistention, thus increasing the "pendelluft" effect. However, they were unable to find a diagnostic method through which the viscoelastic effect could be differentiated from the increase in heterogeneity. A ventilatory strategy with high TVs was used in this study.

In line with previous results, in 1995, Pelosi et al.,^(5^ assessed the effect of PEEP on respiratory resistance in healthy subjects and in patients with moderate/severe ARDS. The subjects without respiratory disease were subjected to 0, 5 and 10cmH_2_O PEEP, whereas the patients with ARDS were subjected to 0, 5, 10 and 15cmH_2_O PEEP. Again, in addition to differences from healthy subjects, patients with ARDS showed an increase in Rrs with the increase in PEEP levels, with significant differences between 10 and 15cmH_2_O PEEP. In this case, similarly to the previous study, the authors speculated that the increase in DRrs reflected changes in stress relaxation and the increase in inequalities in time constants typical of overdistension due to TV, whereas high PEEP values could increase the airway diameter, thus decreasing Rinit. The different magnitude of the changes in each component when increasing PEEP implies that the increase in Rrs reflects the increase in DRrs. In this case, high TVs were also used.

Last, Blanch et al.,^(6^ in 1999, assessed the effect of PEEP on volumetric capnography and respiratory mechanics variables. They analyzed 3 groups: 8 healthy subjects referred for surgery with no history of tobacco smoking, obesity or cardiac disease, 9 patients with acute lung injury and 8 patients with ARDS according to The American-European Consensus Conference.^(11^ All patients were subjected to 4 PEEP levels (0, 5, 10 and 15cmH_2_O). The authors found significant differences in Rrs, DRrs and Rinit both among groups and among PEEP levels. In line with previous studies, DRrs increased as PEEP levels increased, which was reflected in Rrs, whereas Rinit decreased as PEEP levels increased. In this case, volumetric capnography prevented extending the conclusions of previous studies because this technique reflects changes in both ventilation and perfusion, without differentiating the incidence of the increase in stress relaxation or the pendelluft effect. The ventilatory strategy did not differ from that used in the evidence described above: TVs were high. Both in the study by Pelosi et al.^(5^ and in the study by Blanch et al.,^(6^ Rmin decreased with the increase in PEEP levels. This would be explained by the increase in anatomic dead space,^(12^ thereby decreasing frictional resistance to flow.

The main differences from our study were in terms of the ventilatory strategy used. The TV implemented was 382 mL, which represented 5.8mL/kg predicted weight. Setting a lower TV weakened the effects on stress relaxation and lung heterogeneity, and thus, the values were lower than those in the aforementioned studies. Accordingly, the patients in our study experienced a decrease in Rrs with the increase in PEEP levels, that is, when using high TVs, the magnitude of the effect on DRrs ultimately prevailed over changes in Rrs. This phenomenon could be explained by the changes PEEP causes in lung elastance: at higher PEEP levels, lung elastance, overdistension, lung heterogeneity and stress relaxation are higher. In turn, when the TV is low, the DRrs effect is weaker than the effect generated by the increase in PEEP on airway overdistension, thereby decreasing Rrs.

This study had limitations. First, we did not assess the effect of PEEP on different Rrs components, although this was a retrospective analysis of a database collected prospectively. Second, the PEEP levels were gradually but not randomly decreased.

## CONCLUSION

In conclusion, unlike the reported evidence, when using a low tidal volume strategy, the maximum resistance of the respiratory system decreases as PEEP levels increase, reflecting the minimum resistance response, whereas differential resistance increases as PEEP levels increase.
